# Supplementary material for: The interactome of intact mitochondria by cross-linking mass spectrometry provides evidence for coexisting respiratory supercomplexes
Source: Mol Cell Proteomics. 2017 Dec 8;17(2):216–32. doi: 10.1074/mcp.RA117.000470 (PMC5795388; doi:10.1074/mcp.RA117.000470)
Supplement: Supplemental Data [file supp_RA117.000470_133922_0_supp_23978_fzffwf.zip › spectra_annotation/mito_spectra_annotation/22-1-3-1-11-1.pdf]

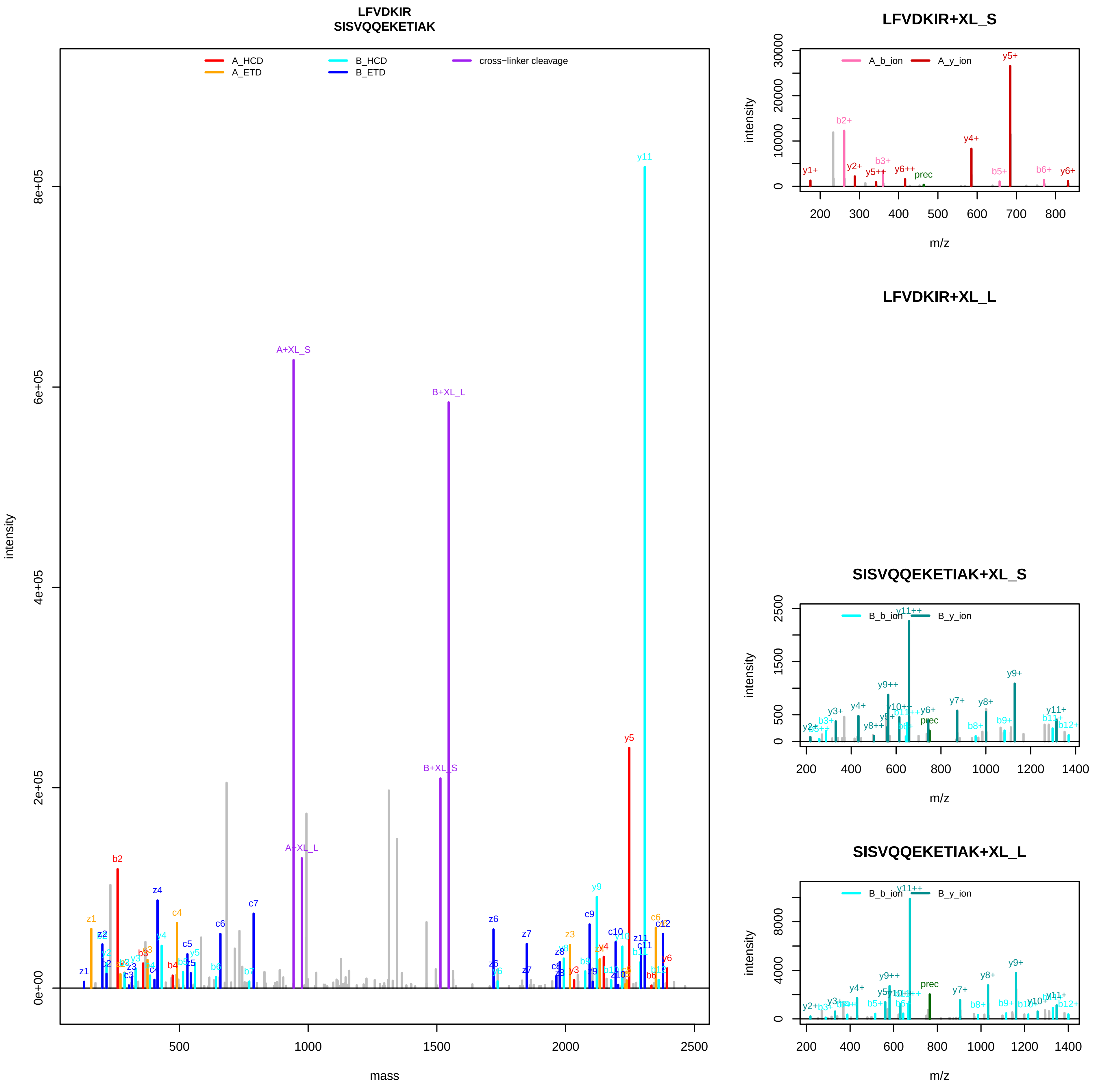

LFVDKIR
SISVQQEKETIAK
A_HCD
A_ETD
B_HCD
B_ETD
cross−linker cleavage
intensity
mass
0e+00
2e+05
4e+05
6e+05
8e+05
500
1000
1500
2000
2500
y11
A+XL_S
B+XL_L
B+XL_S
A+XL_L
y5
b2
z4
c7
c6
z6
z7
c9
y9
c10
c12
c6
z1
c4
z2
LFVDKIR+XL_S
A_b_ion
A_y_ion
m/z
y5+
b2+
y4+
b3+
y2+
y5++
y6++
prec
y1+
b5+
b6+
y6+
LFVDKIR+XL_L
SISVQQEKETIAK+XL_S
B_b_ion
B_y_ion
y11++
y9+
y9++
y7+
y8+
y4+
y3+
y11+
SISVQQEKETIAK+XL_L
